# Engaging stakeholders to strengthen support for community-engaged research at Stanford School of Medicine: An institutional assessment and action planning approach

**DOI:** 10.1017/cts.2025.8

**Published:** 2025-01-24

**Authors:** Patricia Rodriguez Espinosa, Anisha I. Patel, Starla Gay, Ysabel Duron, Alyce S. Adams, Nina Wallerstein, Ruth O’Hara, Lisa G. Rosas

**Affiliations:** 1 Department of Epidemiology and Population Health, Stanford University School of Medicine, Stanford, CA, USA; 2 Office of Community Engagement, Stanford University School of Medicine, Stanford, CA, USA; 3 Department of Pediatrics, Stanford University School of Medicine, Stanford, CA, USA; 4 Black Ladies Advocating for Cancer Care, Oakland, CA, USA; 5 The Latino Cancer Institute, San Jose, CA, USA; 6 Stanford Health Policy, Stanford University School of Medicine, Stanford, CA, USA; 7 Center for Participatory Research, University of New Mexico, Albuquerque, NM, USA; 8 Department of Psychiatry and Behavioral Sciences, Stanford University School of Medicine, Stanford, CA, USA

**Keywords:** Community-engaged research, health equity, institutional assessment, capacity building, community-based participatory research

## Abstract

**Introduction:**

Despite the central role that patient and community engagement plays in translational science and health equity research, there remain significant institutional barriers for researchers and their community partners to engage in this work meaningfully and sustainably. The goal of this paper is to describe the process and outcomes of Engage for Equity PLUS at Stanford School of Medicine, which was aimed at understanding and addressing institutional barriers and facilitators for community-engaged research (CEnR).

**Methods:**

A Stanford champion team of four faculty and two community partners worked with the University of New Mexico team to conduct two workshops (*n* = 26), focus groups (*n* = 2), interviews with leaders (*n* = 4), and an Institutional Multi-Stakeholder Survey (*n* = 35). These data were employed for action planning to identify strategies to build institutional support for CEnR.

**Results:**

Findings revealed several key institutional barriers to CEnR, such as the need to modify organizational policies and practices to expedite and simplify CEnR administration, silos in collaboration, and the need for capacity building. Facilitators included several offices devoted to and engaging in innovative CEnR efforts. Based on these findings, action planning resulted in three priorities: 1) Addressing IRB barriers, 2) Addressing barriers in post-award policies and procedures, and 3) Increasing training in CEnR within Stanford and for community partners.

**Conclusions:**

Addressing institutional barriers is critical for Academic Medical Centers and their partners to meaningfully and sustainably engage in CEnR. The Engage for Equity PLUS process offers a roadmap for Academic Medical Centers with translational science and health equity goals.

## Introduction

Meaningful engagement of diverse patients, community partners, and other key stakeholders is essential for academic medical centers (AMCs) to advance the goals of translational science and health equity. With support from the National Center for Advancing Translational Sciences, AMCs are focused on the goals of translational science to accelerate the transformation of research into improvements in health for the patients they serve as well as their local communities and beyond [1,2]. As part of this focus, AMCs are increasingly prioritizing health equity, the state in which all people have a fair and just opportunity to attain their highest level of health [3,4]. Translational science and the pursuit of health equity both require the involvement of those with lived experience to maximize the potential for effective, sustainable, and scalable solutions [5,6].

Community-engaged research (CEnR) refers to the engagement of patients, community partners, and other key stakeholders in the research process and spans a spectrum of intensity ranging from outreach and consultation to shared leadership and power [7,8]. The goal of engagement activities with lower intensity is to raise awareness about research and obtain feedback on aspects of a research study, such as recruitment strategies, outcomes, and dissemination activities. At the other end of the spectrum, patients and community partners are equal partners in the entire research process from defining the research question to dissemination of findings. Community-based participatory research (CBPR) is aligned with this high level of engagement and emphasizes shared leadership and power [9,10]. In CBPR, researchers and patients or community partners work together to identify a research question, design the study, obtain funding, implement the study, and analyze and disseminate findings. In this manuscript, CEnR is used as an umbrella term for approaches that incorporate groups outside of the university, most notably CBPR and patient-centered outcomes research.

CEnR is well aligned with the goals of translational science and the pursuit of health equity. Patients and communities can take an active role in setting research priorities with the greatest chance of impact based on their lived experience with the conditions that research aims to improve. They can also partner in designing feasible studies that resonate with the patients and communities that researchers seek to engage, thus facilitating recruitment and retention that is representative of the target population – a fundamental goal of translational science. Additionally, when patients and communities, including advocates and policymakers, contribute to analysis and dissemination activities, they can accelerate the translation of findings into practice and policy.

Despite the central role that CEnR plays in translational science and health equity research, there remain significant structural and systemic challenges. Researchers face barriers such as limited resources for forming and nurturing partnerships, long timelines for developing partnerships, and a lack of acknowledgment of CEnR efforts in the AMC promotion process [11–13]. Additionally, community-engaged researchers face unique administrative and financial burdens related to adding community partners and sites to IRB protocols, executing sub-award contracts, and paying community partners in a timely fashion [14]. Patient and community partners also face barriers such as difficulty finding researchers aligned with their community-driven goals, lack of funding to support their involvement, and timelines that do not support their community needs and priorities [15]. Barriers and challenges at the institutional level have been less explored, yet remain major impediments to successful CEnR.

The goal of this paper is to describe the process and outcomes of a project known as Engage for Equity PLUS (E^2^ PLUS) at Stanford School of Medicine that was aimed at understanding and addressing institutional barriers for CEnR at an AMC with deep roots in basic science research and more recent history and systematic investments in purposeful CEnR. We describe the process we used to identify institutional barriers to CEnR and propose solutions to overcome these barriers.

## Methods

### Setting

This project was conducted at Stanford School of Medicine, located in Santa Clara County, California’s Silicon Valley. The region is characterized by stark health inequities driven by wealth disparities and a high cost of living. For example, in 2022, the top 10% of households held 66% of the wealth in Silicon Valley and eight individual residents held more wealth than the bottom 50% combined [16]. Although the poverty rate according to U.S. national standards is low, approximately one-third of households in Silicon Valley reported not earning enough money to meet basic needs, such as housing and food, due to the high cost of living [16]. These issues disproportionately impact the health of communities of color. For example, the prevalence of diabetes in East San Jose, a predominantly low-income Latinx immigrant community, is twice that of Palo Alto, where Stanford is located and is predominantly high-income and non-Latinx white [17].

Stanford School of Medicine has a large biomedical research portfolio with over $500 million annually in grant funding from the National Institutes of Health. The School of Medicine includes several newly established groups that support CEnR activities including the Office of Community Engagement, which is supported by the Clinical and Translational Science Award (CTSA) from NCATS, the Office of Cancer Health Equity, which is supported by the National Cancer Institute Comprehensive Cancer Center Grant, and the Office of Community-Engaged Research of the Maternal and Child Health Research Institute. These community engagement offices were recently established, and new leaders were established for each office within the last five years. The leaders of the CTSA, the Stanford Cancer Institute, and the Maternal and Child Health Research Institute were supportive of the E^2^ Plus process.

### Engage for equity PLUS process

**Figure 1.**
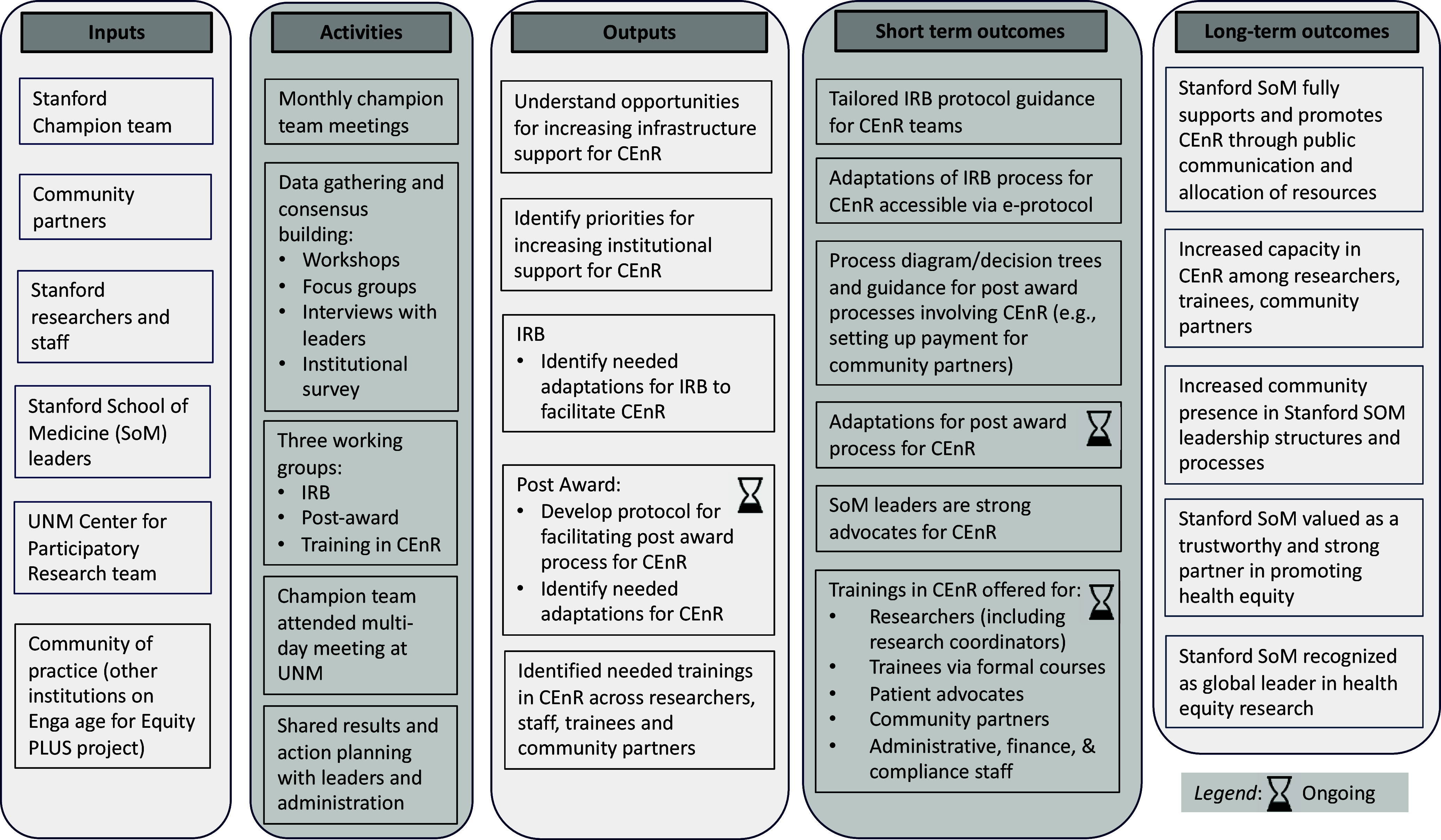
Logic model of Engage for Equity Plus at Stanford School of Medicine. Note: IRB: Institutional Review Board; CEnR: Community-engaged Research; UNM: University of New Mexico.

The University of New Mexico (UNM) partnered with community engagement groups in the School of Medicine to implement the E^2^ PLUS process [18]. The long-term objective of the UNM E^2^ PLUS award was to strengthen institutional support for CEnR. The E^2^ PLUS process was led by a Stanford champion team. The champion team worked with the UNM team to conduct workshops, focus groups, and an institutional survey (Figure 1). The UNM team supported the Stanford champion team in using the data for action planning to identify strategies to build institutional support for CEnR. Finally, the Stanford champion team also met with champion teams from two other institutions also applying the E^2^ PLUS process at their respective institutions. This provided an additional community of practice and layer of support throughout the project.

### Champion team

The Stanford champion team was composed of four faculty from the aforementioned community engagement groups within the School of Medicine (Office of Community Engagement, Stanford Cancer Institute Office of Cancer Health Equity, and the Maternal and Child Health Research Institute’s Office of Community-Engaged Research). The champion team also included two community partners who direct cancer advocacy and survivorship groups or community organizations and who have been active members of CBPR projects at Stanford and were familiar with the institutional capacities and challenges. The faculty members and community partners had worked together previously. Additionally, two of the faculty members had longstanding relationships with the UNM team. During the first year, the UNM team met with the champion team regularly to support their identified targets of action and to provide summaries of data to help the team further their agendas for change and offer any needed technical support.

### Activities and data gathering

#### Workshops

The UNM team, in close collaboration with the Stanford champion team, planned and conducted two workshops to facilitate a visioning process using the CBPR conceptual model to identify common short-term and long-term outcomes [19]. The first workshop occurred in October 2021 with 5 hours across two days. Using E^2^ tools such as the River of Life exercise [20], the UNM team facilitated discussions to acknowledge and recognize the history, context, facilitators, and barriers for CEnR at Stanford. The River of Life is a CBPR tool that allows teams, projects, and partnerships to reflect and document their CEnR journey and to specifically acknowledge major milestones, barriers, and facilitators and use them to project into current and future goals [20]. This was followed by a “Visioning with the CBPR Model” activity to have participants identify and prioritize initial targets of action to strengthen equity-based CEnR at Stanford. A second workshop was held in March 2022 aimed at reviewing data from the prior workshops, as well as focus group data, and further brainstorming and action planning around targets of change identified in the prior workshop.

#### Focus groups and interviews

The UNM team conducted two focus groups, one with Stanford researchers and one with community partners or patient advocates, to better understand their needs around CEnR, as well as similarities and differences among these stakeholder groups. They recruited participants for the focus groups from the workshop attendees. The UNM team also conducted one-on-one interviews (*n* = 4) with Stanford leaders who oversaw the community engagement groups, including the PI of the CTSA, a clinical Department Chair, a Center Director, and leaders of Stanford-wide efforts on justice, equity, and inclusion. Leaders were identified by the Champion team based on their overall institutional knowledge, leadership on community engagement and health equity, and having leverage to support the implementation of changes identified as part of this project. The focus groups and interviews were conducted over Zoom, audio recorded, and transcribed verbatim. The UNM team conducted the formal analysis and reported themes to the Stanford champion team. Full methodological details are provided elsewhere [21].

#### Institutional multi-stakeholder survey

The newly developed and novel Institutional Multi-Stakeholder Survey [22] was sent to institutional leaders, academic investigators, and community partners involved in CEnR at Stanford Medicine. The survey assessed domains such as institutional CEnR policies/practices, community processes/structures, the function of formal Community Advisory Boards (CABs), climate/culture for CEnR, and perceptions of institutional leadership for CEnR. The Stanford champion team offered leadership and support in adapting the survey for the Stanford context (e.g., ensuring that various offices that support community engagement, the CTSA, the Cancer Institute, and other relevant Centers on campus were reflected in the questions’ introductions). The champion team also supported by sending personalized invitations and reminders to respondents; however, the survey was administered by the UNM team.

### Action planning

The champion team met monthly throughout the project, which included action planning. A member of the UNM team attended the meetings to support action planning. The UNM team provided summaries of the data analysis of the workshops, focus groups, and interviews, as well as the institutional survey. The champion team used these data to identify key institutional barriers to CEnR. The champion team also used the data to identify feasible and impactful solutions to propose for each barrier. The champion team worked with institutional leaders and key stakeholders to begin implementing solutions to increase institutional support for CEnR.

## Results

### Workshops

**Figure 2.**
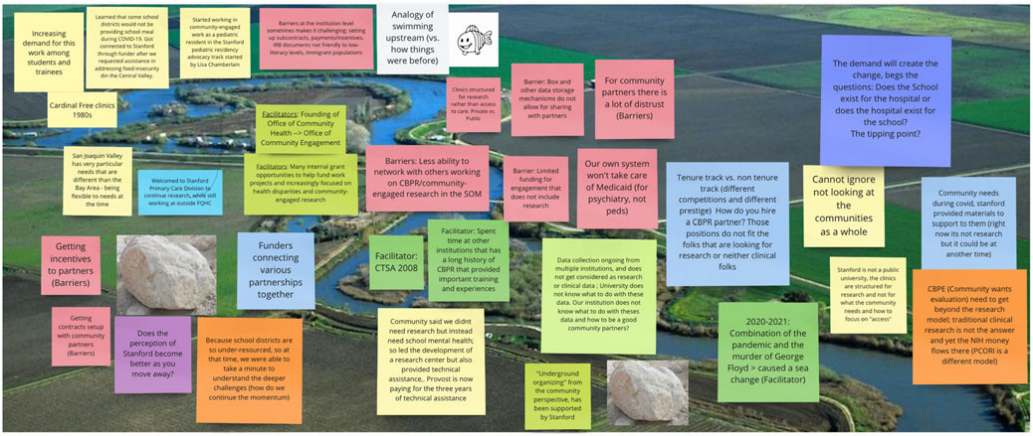
River of life reflection process on Community-engaged research in Stanford School of Medicine. Miro board developed by one of three small groups in the Fall workshop. CTSA: Clinical and Translational Science Award; PCORI: Patient-Centered Outcomes Institute; CBPE: Community-Based Participatory Evaluation.

Workshops included a total of 26 attendees, including Stanford faculty and staff (*n* = 15) across various departments and ranks and community/patient members (*n* = 11). Each workshop lasted approximately 2.5 hours. The main outcome of the first workshops was a reflection, via the River of Life exercise, on the School of Medicine history of CEnR, including a rich history of community clinical service and the establishment of free community clinics since the 1970s, with an emphasis on medical education and service learning (Figure 2). Attendees also discussed how recent events, including COVID-19 and the murder of George Floyd further synergized efforts to support and expand CEnR and health equity research. The establishment of the CTSA, the Cancer Institute, and relevant offices supported by both awards with missions around advancing CEnR and fostering sustainable bidirectional partnerships were also cited as important facilitators. Attendees also began discussion and prioritization of key barriers including IRB and post-award challenges and identified both as key targets for change.

**Figure 3.**
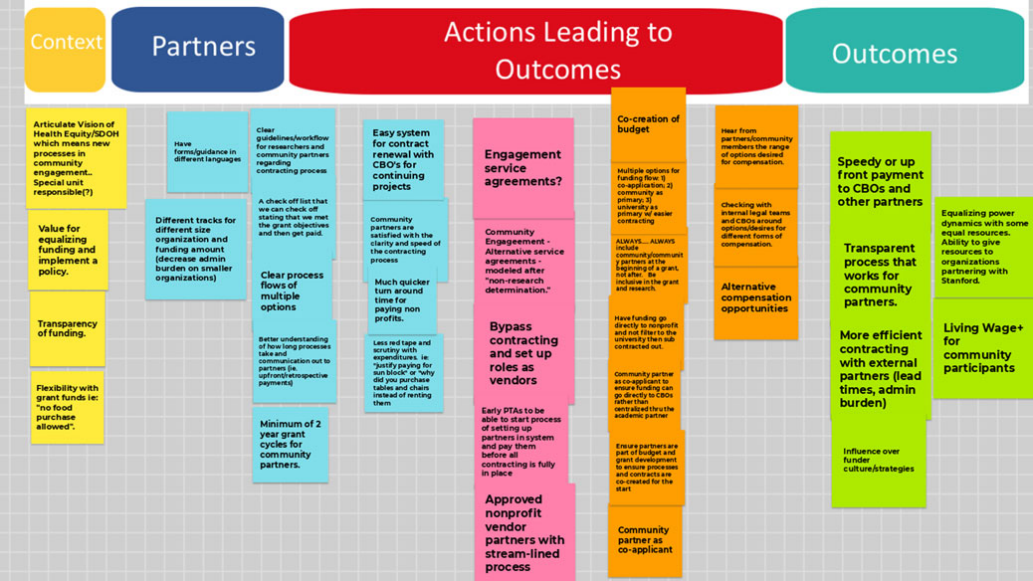
Small group discussion on post-award barriers and proposed solutions from Engage for Equity PLUS workshop in Stanford School of Medicine. Notes: CBO: community-based organizations.

The main outcome of the second workshop was further discussion of challenges with a concentration on the identification of short- and long-term actions relevant to solutions and institutional practices to support further CEnR (Figure 3). Two working groups, related to IRB and post-award barriers, respectively, were recommended. An additional priority area around training was also identified.

### Focus groups and interviews

The focus groups (*n* = 2) with community partners (*n* = 4) and with researchers (*n* = 4), as well as interviews with leaders (*n* = 4) revealed several key institutional barriers to CEnR at Stanford, as well as recommendations for improvement (Table 1). Similar to workshop discussions, participants initially acknowledged the history of emphasis on basic science and the comparative more recent investment in CEnR. For immediate short-term action, researchers and representatives of community-based organizations typically advocated for modifying existing organizational policies and practices to expedite and simplify community-based research administration and payment for community partners engaged in research with Stanford.

**Table 1.** Summary of qualitative findings from focus groups with researchers and community partners and interviews with leadership, Engage for Equity PLUS, Stanford CA

Main finding	Exemplary Quotes
Tension between historic institutional emphasis on excellence in basic science research and more recent emphasis on health equity placed-based research	“Stanford’s focus on basic science and international prestige as a private university, has made the institution far removed from the communities that we come from or we represent.”
Working towards increased institutional trustworthiness is a desired outcome by academic and community partners alike. Yet, the culture and climate could be perceived as exclusionary due in part to barriers to accessing healthcare.	“The public doesn’t know distinction between Stanford School of Medicine and Stanford Medicine (hospital) – so both often perceived as exclusionary, in part because healthcare system isn’t as accessible to many publicly insured (Medicaid) patients.”
Need to extend partnership networks to mitigate burnout of highly engaged community partners. Involving the same partners can also lead to potential bias in whose voices are represented and limits the opportunity for multiple stakeholders to collaborate with researchers to support and evaluate their own broader health equity initiatives.	“Asking the same people to come to the community advisory table all the time. […] One will refer their friend to the same person and the same person and the same person and this is where we talk about burnout of community activists who will be there because they want the voice at the table.” “We can talk and say “oh you know we need data and we need to take a look at this and we need to take a look at that.” However, unless you are already in touch with the research institutions that you will most likely have to go to for these things, you have no idea if you are not already connected.”
Need to revisit traditional academic systems and practices such as the IRB, contracting, and compensation for external research partners, as well as tenure and promotion guidelines	“IRB process is based on lab or basic science research” “Research is very narrowly defined in this institution, as is scholarship. Community commitment and collaboration are not given the same weight in promotion or hiring. Faculty in the community engagement track are not seen as equal to the ‘research’ faculty and do not receive many of the same benefits. It is a non-equitable structure based on a biased archaic academic hierarchy.”
Fragmentation of current Community-engaged Research (CEnR) efforts across campus.	“We have PIs, faculty, all over the organization, and when I say organization now, I mean Stanford University not just the School of Medicine, who are doing research related to disparities, health disparities, doing research related to health equity, and those faculty members very rarely, if ever, talk to each other.” “My observation is that we can still be a bit patchwork quilt about our approach; we do a little bit here, more there. However, we need, I think, a more defined, systematic approach to the science of community engagement and the action and implementation of community engagement across every domain of our research enterprise.”
Some leaders advocated for the development of greater institutional connections between Community-Based Participatory Research (CBPR) and policy-oriented investigators, think tanks, advocates, and policymakers to push policy reform.	“We also want to look at the translational aspect…Having individuals with policy expertise to pull in the advocates when needed and get the questions answered that advocates need to move on policies on the state or local level. Getting the information to the policymakers before the research is published is also extremely important.”
Need for workforce and leadership development was emphasized as mid-to-long-term strategy to increase institutional capacity in CEnR	“Cultivate internal leadership development in CBPR and policy research by creating mid-management positions in departments and centers throughout the School of Medicine.”

Silos in the work were also noted by participants, with leaders further elaborating that the lack of collaboration between research groups creates missed opportunities to form interdisciplinary research teams that interface with a variety of community stakeholders. Leaders perceived that this represents a missed opportunity to tackle health inequities more effectively.

Capacity building, both via training, as well as recruitment and development of faculty and leaders with expertise in CEnR was also identified as a target outcome. According to one top leader, systematically supporting CEnR efforts “requires more definition and more education of our faculty who don’t traditionally see this as a necessary component for their research.”

Despite noted barriers and opportunities for improvement, focus groups and interview participants consistently noted that the Department of Pediatrics and the community engagement cores at both the CTSA and the Cancer Institute offer leadership and innovation in CEnR efforts. All three groups were commonly cited as engaging in promising practices that could be further disseminated to other divisions and departments to increase CEnR capacity as well as the number of investigators and trainees who adopt this research approach in their own work.

### Institutional multi-stakeholder survey findings

Thirty-five individuals completed the Institutional Multi-Stakeholder Survey including 14 academic researchers (faculty, researchers, institutional leaders), 8 academic staff from offices supporting CEnR, and 13 community partners representing community-based organizations or other local agencies, patient advocacy groups, and CAB members. Notably, across the majority of the survey domains, one-quarter to one-third of respondents reported that they did not know, with the exception of the domains for the function and priorities for the Community Advisory Boards (CABs) and the institutional climate and culture regarding CEnR (Table 2; additional survey findings are shown in the Supplemental Materials. Disaggregated information and survey psychometrics are discussed in Dickson et al [22]). In response to items in the CEnR policies and practices domain, 49% of respondents reported “not at all” or “slightly” when asked if Stanford School of Medicine supports involvement of patient and community partners in all stages of research. These responses were driven primarily by academic partners, 70% of whom endorsed this response option, compared to only 15% of community partner respondents. Another 21% of respondents reported that they did not know about such policies and practices. Half of respondents (50%) indicated that community members are not at all or slightly involved in strategic planning for the University. Academic partners were more likely to endorse “not at all” or “slightly” response options for this item (65%) compared to community partner respondents (25%). An additional 28% reported that they did not know if community members were involved in strategic planning. Among community partner respondents, 50% selected the “do not know” option for this item.

**Table 2.** Institutional multi-stakeholder survey finding from Engage for Equity PLUS, Stanford CA

Domain	Response options			
**Policies and Practices**				
*How much is each policy or practice applied at Stanford School of Medicine?*	Not at all/Slightly	Moderately	Often/All the time	Don’t know
Hiring and training patient and community partners as research coordinators/staff	46%	12%	15%	27%
Supporting involvement of patient and community partners in all stages of research (question to results)	49%	9%	21%	21%
Community Engagement products in tenure and promotion guidelines for faculty	41%	9%	9%	41%
IRB policies and practices that support CEnR	56%	13%	6%	25%
Offering education for partners on research processes	47%	15%	16%	22%
Having community input in strategic planning at leadership level	50%	16%	6%	28%
**Community Advisory Board (CAB)**				
*Please consider to what extent the CAB at your Office/Center of Community Engagement engages in the following practices:*	Not at all/To a small extent	To a moderate extent	To a great/To a complete extent	Don’t know
Identifies research needs and priorities	46%	27%	9%	18%
Consults on cultural issues	18%	0%	73%	9%
Strengthens collaborations between academic and community/patient partners	18%	36%	37%	9%
Develops plans for using findings to benefit the community	46%	0%	46%	8%
**Climate/Culture for Community-Engaged Research (CEnR)**				
	Strongly/ somewhat disagree	Neither agree nor disagree	Somewhat/Strongly agree	Don’t know
Researchers and staff are willing to change how we conduct research in response to community or patient advocate feedback	25%	0%	70%	5%
We regularly take time to reflect on how we are working towards equity	25%	10%	60%	5%
Your Office/Center of Engagement contributes to developing programs and interventions with community partners to address conditions that affect health inequities.	20%	10%	70%	0%
Your Office/Center of Engagement has the necessary budget or financial resources to support patient- and community-engaged research	40%	15%	35%	10%
Your Office/Center of Engagement has the necessary training resources to support patient- and community-engaged research	45%	5%	35%	15%
**Perception of Leadership Engagement**				
	Strongly/ somewhat disagree	Neither agree nor disagree	Somewhat/Strongly agree	Don’t
Ensures researchers and staff have resources necessary to conduct CEnR	44%	6%	19%	31%
Strongly supports training and development of community-engaged scholars	48%	7%	19%	26%
**Advocacy for Community or Patient-Engaged Research**				
	Strongly/ somewhat disagree	Neither agree nor disagree	Somewhat/Strongly agree	Don’t know
I am, or researchers I work with, are able to reflect and identify systems of power that influence treatment of community members and patients	10%	19%	61%	10%
I am, or the researchers I work with, are comfortable developing an action plan to confront barriers to health equity that impacts community members and patients.	6%	22%	59%	13%

*Note*. Similar response options were combined for presentation purposes.

The responses in the domain for CABs varied widely, with a significant proportion of respondents endorsing infrequent engagement of CABs across the various items and another significant proportion endorsing that CABs were engaged to a great or complete extent. For example, in response to the item “Develops plans for using findings to benefit the community,” 46% reported “not at all” or “to a small extent” and 46% also reported “to a great or complete extent.” Notably, 100% of community partners endorsed “to a great” or “to a complete extent” for this item. Consulting on cultural issues was the most endorsed activity for the CAB among respondents, with most of academic and community partners selecting “to a great” or “to a complete extent.” For this domain, academics more frequently endorsed “do not know” response options compared to community partners.

In the climate and culture domain, respondents highlighted positive attributes and also resource needs. For example, 70% of respondents reported that they somewhat or strongly agreed that Stanford researchers and staff are willing to change how they conduct research in response to community or patient advocate feedback. Both academics and community partners favorably endorsed this item (77% and 57%, respectively). Similarly, 70% (85% academic partners and 43% of community partners) reported that the specific office or center that they work with contributes to developing programs and interventions to address conditions that affect health inequities. In terms of resource needs, 40% reported that they had the necessary budget or financial resources to support CEnR and 35% reported that they had the training resources to support CEnR, which were endorsed similarly among academic and community partners.

Respondents were mixed in their perception of leadership support, with some strongly or somewhat disagreeing that researchers and staff have the resources necessary to conduct CEnR (44% overall; 60% and 17% for academic and community respondents, respectively) and support the training and development of community-engaged scholars (48% overall; 65% and 18% for academic and community respondents, respectively). Another proportion of respondents somewhat or strongly agreed that leaders ensure resources (19%) and support training (19%). Community partner respondents were more likely to indicate “do not know” for items in this domain.

In terms of advocacy, approximately two-thirds of respondents somewhat or strongly agreed that researchers are able to reflect and identify systems of power that influence the treatment of community members and patients (61% overall; 74% and 42% for academic and community respondents, respectively) and are comfortable developing an action plan to confront barriers to health equity that impact community members and patients (59% overall; 70% and 42% for academic and community respondents, respectively). Slightly fewer (44%) strongly or somewhat agreed that researchers are able to negotiate for equitable services and research on behalf of the community members and patients (shown in Supplemental Materials).

Finally, the survey also highlighted key strengths, such as the commitment of individual researchers, staff, and community engagement offices to the principles of effective CEnR, including a willingness to incorporate community input into their research and practices. Moreover, a commitment to advocacy for patient and community partners and to promoting health equity was also showcased.

### Action planning

Data gathered from the various stakeholders and using different approaches converged in support of the importance of addressing institutional policies and procedures (i.e., IRB and post-award financial processes) that can further support the use of CEnR in health equity and translational research. Finally, leaders, researchers, and community stakeholders all agreed that creating training opportunities for faculty and community/patient partners was essential to CEnR at Stanford. Based on these findings, the champion team action planning resulted in three priorities for strengthening institutional support for CEnR: 1) Addressing IRB barriers, 2) Addressing barriers in post-award policies and procedures, and 3) Increasing training in CEnR within Stanford and for community partners (Table 3).

**Table 3.** Barriers and proposed solutions by the three priority areas from Engage for Equity PLUS, Stanford, CA. Proposed solutions are indicated as “being in development*,” and “currently being implemented or completed**” by the asterisk; those with no asterisks have not yet been started

Barriers	Proposed solutions
**Institutional Review Board**	
Additional training is needed for Institutional Review Board (IRB) members on community-engaged research to enhance the review process and better support academic-community partner teams.	Provide training for IRB members and research teams on protections for human subjects in community-engaged research. Ensure Community Advisory Board and other similar groups can liaison with the IRB and influence decisions. Moreover, have opportunities for Community Advisory Board members to serve on the IRB panels, if desired. Offer training for community or patient advocate partners to serve on the IRB and further learn about human subject research and its oversight.
IRB forms and templates are difficult to adapt for community-engaged research. Current forms seem too complex for populations that have limited literacy.	Adapt new forms and templates to reflect the nature of community-engaged research. Build on templates from other institutions, as well as other Stanford templates for non-medical research, that are designed specifically for community-engaged research.* Collaboratively review forms with community partners to ensure relevant language and literacy requirements.
IRB consent forms include language about the need to document social security numbers/tax id numbers for providing participant payments. This is a barrier for working with undocumented immigrants and similar communities.	Policy changes related to social security number/tax id number language in consent forms for payments that do not exceed a certain amount.
The Collaborative Institutional Training Initiative training is not accessible for community partners, which is needed to ensure that partners have the capacity to engage in human subjects research.	Formalize the availability of alternative approved IRB trainings on the website and ensure that multiple languages are offered.* Provide trainings and conduct marketing efforts to make sure the whole community is aware.
It is difficult to indicate the role of a community partner in the IRB application. For community-engaged research, the partner plays a central role. However, from the perspective of the IRB this may not be considered a central role.	Toolkit of options for IRB process for community-engaged researchers. More clarity and examples of when and how to list partners (e.g., multi-site vs. collaborator) would be helpful.*
There is currently no process for outside partners to rely on the Stanford IRB when seeking approval for new protocols.	Discuss reliance processes with IRB and make policies clear to researchers and community partners.* If external IRB is required by the Stanford IRB, set clear expectations regarding what steps will still require IRB approval and provide guidance for reconciliation across IRBs if the Stanford PI and Community PI are covered under different entities.*
**Post-award process**	
The length of time for getting a contract in place and making payments is a burden, especially for small community-based organizations that do not have the funds to work until the funding is made available.	Decision trees and clear instructions for different administrative processes related to paying community partners – process maps for end user including responsibilities and roles of researchers and community partners – Standard operating procedures with detail of the reasoning behind each step and policy.* Workflow diagram to help teams find out which type of agreement is needed and the typical length of time to completion.*
The administrative burden for small organizations is very high. The same process that is required for large academic institutions is expected for small community-based organizations and they do not have the infrastructure to successfully get through the process.	Adapt procedures for the size and scope of the project and community partner. Explore the feasibility for an expedited process for smaller organizations/projects. Assign a research team member or administrative support staff who can be assigned to work on post-award contracts with small community partners. For seed grants, make payments directly to community partners as opposed to provide all the money to the Stanford researcher.
Challenges exist with funders, including lack of funding for food and other culturally relevant items often needed in health equity and CEnR. Moreover, funding timelines, and administrative burden, often limit community partner ability to participate and hinder academic-community partnership development and sustainability.	Use institutional power and resources to influence funders. Explore different timelines for CEnR projects, including different timelines for community partners. Reduce administrative burden and technology requirements (e.g., related to submission and progress reports) to facilitate engagement of smaller community-based organizations and grassroots partners. Explore alternative mechanisms of making funds available to community partners, instead of funding always being managed by academic partner.*
**Training in Community-engaged Research (CEnR)**	
Need for training for both researchers and community members/patient partners in CEnR. Lack of a central location for supporting documents and materials (e.g., sample budgets, evaluation tools, guides for supporting community advisory boards) for researchers and community members to access.	Develop variety of trainings (formal courses, workshops, others) for faculty, trainees, and research coordinators, on CEnR, Community-Based Participatory Research (CBPR), and patient-centered outcomes research, and best partnering practices throughout the research process.** Identify needs and develop variety of trainings for community and patient partners to build research capacity. Collaborate with Community Advisory Boards and other partners to identify topics and format. Ensure partners are compensated for their time and that trainings are accessible across various languages.** Include seed funding for community partners to start a partnered research project following their training.** Compile and make accessible to researchers and community/patient partners, in multiple languages and formats, relevant materials to support grant writing and research relevant to CEnR.** Implement technical assistance portal, triaged by various offices on campus supporting CEnR, to support both academic and community partners in various aspects of the research process, from study design and funding acquisition to implementation and evaluation.**

*Note*. Barriers and solutions were further developed during action planning stages.

The champion team presented these priorities to School of Medicine leadership, including at a meeting for all School Department Chairs, with the leader of the IRB, and with the leader for the CTSA. The champion team also developed specific activities to advance the work. Some activities were implemented in the short-term while other activities, particularly those requiring substantial resources, funding, or coordination among multiple groups across the University, will take longer to implement. For addressing barriers in the IRB process, the champion team reviewed existing literature that summarized barriers and solutions for IRB review of CEnR and met with the IRB lead to discuss the next steps [23]. To address barriers in the post-award process, the champion team worked with the central University finance office to better understand how vendors and subcontracts are established and to share findings from the E^2^ PLUS process. For increasing training, the champion team established two new training programs for patient advocates and community partners, respectively, and an academic track for CEnR in the Department of Epidemiology and Population Health for students and trainees. Tip sheets, resource libraries, and webinar training series have also been launched, including trainings developed for faculty, staff, trainees, and research coordinators.

Additionally, the data underscored opportunities for improvement that were not initially prioritized in the action planning phase but were later addressed. For example, the survey highlighted to leadership the lack of involvement of community and patient partners in strategic planning. The CTSA now includes CAB members in strategic planning and includes CAB members in all high-level meetings. The work to further advance these priorities for strengthening institutional support is ongoing and is further informed by discussions with the CAB.

## Discussion

Using a systematic and multi-method, multi-sectoral, assessment process, we present the approach and findings of a project assessing institutional barriers and facilitators for CEnR within an AMC and working collaboratively to address them. Findings highlighted several opportunities to increase and strengthen institutional support for CEnR and existing strengths that can be further supported. The findings were well aligned with community engagement goals of major funders such as the National Center on Advancing Translational Science for their CTSA awards, the National Cancer Institute for their Comprehensive Cancer Centers, and others [24–26]. Workshop discussions and qualitative research highlighted the importance of taking into account institutional context and changing histories and investment in CEnR, and provided additional context to the quantitative survey data. The Institutional Multi-Stakeholder survey findings underscored a significant need to raise awareness about existing institutional support for CEnR. One-quarter to one-third of respondents endorsed the “do not know” option across the majority of survey items. This may reflect the fact that CEnR is a newly emerging effort at Stanford given that the community engagement offices involved in this project were all established in the last five years.

Consistent with prior studies, processes and policies surrounding IRB and post-award financial and contracting requirements were identified as key barriers to CEnR [14,15,23]. These findings may reflect challenges with community partners not being recognized as equal research partners in the IRB process and the lack of cultural competence and accommodation of varying literacy levels in IRB documents, as identified in other studies [23]. Proposed solutions included providing an addendum to the IRB protocol to describe the role of the community partner(s), including CEnR-trained community representatives on the IRB panels, and providing plain language summaries of IRB documents such as consent forms. Implementation of these recommendations can support existing standards set forth by the Association for the Accreditation of Human Research Protection Programs that require engagement of the community in research (Standard I-4) [27].

Regarding the topic of post-award challenges, particularly issuing timely payments to community partners and establishing contracts, one solution has come from funders of CEnR. For example, the California Breast Cancer Research Program recognizes the shared leadership of CBPR partnerships by providing two contracts for one project with one for the community partner and one for the academic partner. This alleviates any need to establish subcontracts and can facilitate equitable partnership through resource sharing.

Training in CEnR for academic and community partners, as well as for staff members in key offices such as IRB and Offices of Sponsored Research was highlighted as an action item needed to build capacity. This is consistent with CTSA efforts to develop CEnR trainings across AMCs nationwide [28]. However, while most existing CEnR trainings highlighted in the extant literature tend to center on individual or team-level capacity building [28], our findings and action planning also identified and worked on trainings aimed at administrative personnel (including IRB officials) and offices involved in pre- and post-award procedures. Building capacity among leadership and further investing in recruiting faculty and personnel with CEnR expertise were also identified as mid- to long-term actions.

In addition to the data collection as a building block for institutional reflection, our process revealed two important components that contributed to our success: engaging a champion team and having the support and involvement of high-level leaders throughout the process. The champion team brought in-depth knowledge of the barriers, facilitators, and institutional context, and monthly meetings ensured accountability and facilitated ownership for various tasks. Meanwhile, supportive leadership at the levels of the CTSA, Cancer Institute, and Stanford’s Maternal and Child Health Research Institute legitimized the process and facilitated both data collection and action planning and execution. For example, leaders connected champion team members with key decision makers in the IRB and Office of Sponsored Research, presented the process at executive meetings to garner additional support and ideas for process/policy changes, and ensured that the momentum on action steps continued beyond the data collection and dissemination phases.

As previously discussed, findings also revealed the importance of widely and frequently disseminating information on current efforts and resources for CEnR and health equity research in general. This includes training opportunities, seed funding, support in grant writing and evaluation, matchmaking between academics and community partners, and networking with other researchers engaged in CEnR. Moreover, there is a need to disseminate opportunities for researchers and teams to receive input from a variety of CABs, including CABs who advise institutional programs such as our existing CTSA CAB. This CTSA CAB gives input to investigators, as well as to CTSA programs such as the KL2/K12 scholars and others.

In addition to addressing these key barriers to CEnR, the workshops, qualitative data, and survey data highlighted an overarching need for the institution to build trustworthiness through nurturing bidirectional partnerships, making research findings and resources accessible, and demonstrating the value of research partnerships across diverse communities and especially in the local region where Stanford is located. Building trustworthiness is a long-term process that requires significant investment from leadership, coordination among faculty and staff, and co-learning in the context of bidirectional partnerships. The E^2^ PLUS process provided a critical catalyst for the institution and its community partners to reflect on recent progress in building capacity for CEnR and institutional support for health equity research.

While the E^2^ PLUS process provided a systematic approach to documenting and addressing barriers, challenges existed. First, the process was time intensive, requiring support from a diverse team of faculty and community partners, as well as leadership. Members of the champion team had to balance competing priorities and sustain their effort in the project for a significant period, from design and launching, to data collection and synthesis to an extended, and still ongoing, period of action planning and implementation of recommendations. The E^2^ PLUS team provided a community of practice with champion teams at other AMCs to facilitate sharing of strategies to address these challenges. Second, given the primary goal of change at the institutional level, the benefits and burden to community partners had to be managed so that partners could still prioritize their own organizational goals and activities, as well as their existing involvement in CEnR projects or CABs with Stanford. A tangible benefit to community partners was the opportunity to take part in national-level meetings and dialogues about CEnR and the importance of addressing institutional barriers from their perspective, as well as networking opportunities with other community partners and organizations involved in the project, locally and nationally. Third, it was important to ensure that participants, including external partners, understood realistic levels of timeliness and responsiveness for their recommendations. Administrative and policy changes, for example, can take a significant amount of time and go through different levels of approval, before coming to fruition. Moreover, meaningful and sustainable change in policies and practices will ultimately require changes in the practices of funders and accreditation bodies (e.g., those that accredit IRBs) to better support CEnR at AMCs and similar institutions.

This E^2^ PLUS process can serve as a model for other AMCs and institutions for assessing current institutional barriers and facilitators of this work and offer a roadmap for how to use findings to take meaningful and sustainable action. This is consistent with literature supporting the importance of reflection as an evidence-based best practice in CEnR and a key pathway to ensure equitable partnering practices and power sharing [29,30]. While the extant literature has primarily centered on individual and team-level reflection [29,31], our approach can be replicated in AMCs to engage in collective institutional-level reflection aimed at furthering CEnR and health equity research. The process at Stanford may be particularly relevant for institutions that have more recently implemented a focus on CEnR as was the case at Stanford.

### Limitations

This process included several limitations. First, assessments, and hence findings, were tailored for our specific institution and context to obtain specific information to inform action steps relevant to our needs, capacity, and context. Thus, while the assessment and overall process can be generalizable, our specific findings and action planning might not be generalizable to other institutions. Second, our sample size was limited and had an overrepresentation of academic and community partners with significant experience and long-term engagement in CEnR. These partners are intrinsically familiar with historic and current challenges and might not represent the views of all engaging in this work, particularly of newer partners. Moreover, due to their commitment and desire to improve CEnR infrastructure for all, participants might have centered their comments more heavily on challenges and opportunities for improvement. Third, due to the emphasis on institutional assessment and change, this process was very Stanford-focused, which can leave community partners wondering what their role is or what the benefits might be for their organizations or specific projects. Frequent discussions with these partners, including invitations to join champion team meetings, were useful for our team to offer clarification and arrive at a mutual understanding of the benefits of the process for all.

## Conclusions

Addressing institutional barriers to CEnR is a critical step to allow community-university partnerships to effectively pursue their health equity goals. The E^2^ PLUS process provided a systematic approach to documenting and addressing these barriers. For AMCs that have had a biomedical and/or bench science focus, it is important to build infrastructure for CEnR, including optimizing policies and procedures such as IRB and post-award and financial oversight to better support partnerships. Additionally, training and capacity building for community partners, faculty, staff, and trainees are needed so that the institution is ready to leverage partnerships and support workforce and leadership development in CEnR.

## Supporting information

Rodriguez Espinosa et al. supplementary material Rodriguez Espinosa et al. supplementary material

## Supplementary material

The supplementary material for this article can be found at https://dx.doi.org/10.1017/cts.2025.8.
