# Supplementary material for: Engaging stakeholders to strengthen support for community-engaged research at Stanford School of Medicine: An institutional assessment and action planning approach
Source: J Clin Transl Sci. 2025 Jan 24;9(1):e36. doi: 10.1017/cts.2025.8 (PMC11883597; doi:10.1017/cts.2025.8)
Supplement: Rodriguez Espinosa et al. supplementary material [file S2059866125000081sup001.docx]

**Supplemental Materials**

**Table 2, Full Version.** Institutional Multi-Stakeholder Survey Finding from Engage for Equity PLUS, Stanford CA.

| **Domain** | **Response options** | | | | |
| --- | --- | --- | --- | --- | --- |
| **Policies and Practices** |  |  |  |  |  |
| How much is each policy or practice applied at Stanford School of Medicine? | Not at all/Slightly | Moderately | Often/All the time | Don’t know |  |
| Hiring and training patient and community partners as research coordinators/staff | 46% | 12% | 15% | 27% |  |
| Supporting involvement of patient and community partners in all stages of research (question to results) | 49% | 9% | 21% | 21% |  |
| Having policies that require partners to review grant applications for community benefit | 50% | 4% | 15% | 31% |  |
| Sharing information about financial practices or requirements with patient and community partners related to research | 46% | 12% | 8% | 35% |  |
| Providing timely payments to partners for their participation | 42% | 15% | 15% | 27% |  |
| Providing timely payments to community subcontractors | 46% | 12% | 12% | 31% |  |
| Community Engagement products in tenure and promotion guidelines for faculty | 41% | 9% | 9% | 41% |  |
| IRB policies and practices that support CEnR | 56% | 13% | 6% | 25% |  |
| Minimizing barriers to participation for partners in research | 56% | 12% | 8% | 24% |  |
| Having written standards that provide expectations around CEnR | 44% | 12% | 16% | 28% |  |
| Evaluating if staff/faculty meet expectations for CEnR | 54% | 4% | 13% | 29% |  |
| Offering education for partners on research processes | 47% | 15% | 16% | 22% |  |
| Having standards for oral and written information that meets cultural, linguistic, and literacy needs of community | 44% | 20% | 12% | 24% |  |
| Identifying and adapting to changes within communities | 32% | 20% | 20% | 28% |  |
| Having community input in strategic planning at leadership level | 50% | 16% | 6% | 28% |  |
| Having funding strategies to mobilize community partners to research health inequities | 56% | 8% | 12% | 24% |  |
| **Community Processes and Structures** |  |  |  |  |  |
|  | Not at all/To a small extent | To a moderate extent | To a great /To a complete extent | Don’t know |  |
| To what extent are community members engaged in strategic planning at the top institutional level | 56% | 3% | 3% | 38% |  |
| To what extent are patients engaged in strategic planning at the top institutional level | 56% | 3% | 3% | 38% |  |
| **Community Advisory Board (CAB)** |  |  |  |  |  |
| Please consider to what extent the CAB at your Office/Center of Community Engagement engages in the following practices: | Not at all/To a small extent | To a moderate extent | To a great/To a complete extent | Don’t know |  |
| Identifies research needs and priorities | 46% | 27% | 9% | 18% |  |
| Consults on cultural issues | 18% | 0% | 73% | 9% |  |
| Strengthens collaborations between academic and community/patient partners | 18% | 36% | 37% | 9% |  |
| Provides ideas and assistance for recruitment of research subjects or research participants | 18% | 27% | 46% | 9% |  |
| Develops plans for using findings to benefit the community | 46% | 0% | 46% | 8% |  |
| Assists with sustainability planning | 46% | 18% | 18% | 18% |  |
| Assists with strategic planning | 40% | 20% | 30% | 10% |  |
| **Climate/Culture for Community-Engaged Research (CEnR)** |  |  |  |  |  |
|  | Strongly/  somewhat disagree | Neither agree nor disagree | Somewhat/Strongly agree | Don’t know |  |
| Researchers and staff are willing to change how we conduct research in response to community or patient advocate feedback | 25% | 0% | 70% | 5% |  |
| Researchers and staff regularly evaluate together how we've done and how we can improve our work | 20% | 5% | 55% | 20% |  |
| Staff frustration is common | 30% | 20% | 40% | 10% |  |
| We regularly take time to reflect on how we are working towards equity | 25% | 10% | 60% | 5% |  |
| Your Office/Center of Engagement actively mobilizes community partnerships and actions to address the environmental, social, and economic conditions that influence health outcomes. | 25% | 5% | 65% | 5% |  |
| Your Office/Center of Engagement contributes to developing programs and interventions with community partners to address conditions that affect health inequities. | 20% | 10% | 70% | 0% |  |
| Your Office/Center of Engagement contributes to advocating for policies that address conditions that affect health inequities. | 20% | 20% | 60% | 0% |  |
| Your Office/Center of Engagement contributes to new insights, innovative solutions and the evidence base to address health inequities and community conditions that influence health. | 10% | 10% | 80% | 0% |  |
| Your Office/Center of Engagement has the necessary budget or financial resources to support patient- and community-engaged research | 40% | 15% | 35% | 10% |  |
| Your Office/Center of Engagement has the necessary training resources to support patient- and community-engaged research | 45% | 5% | 35% | 15% |  |
| Your Office/Center of Engagement has the necessary staffing resources to support patient- and community-engaged research | 55% | 5% | 20% | 20% |  |
| **Perception of Leadership Engagement** |  |  |  |  |  |
|  | Strongly/  somewhat disagree | Neither agree nor disagree | Somewhat/Strongly agree | Don’t know |  |
| Ensures researchers and staff have resources necessary to conduct CEnR | 44% | 6% | 19% | 31% |  |
| Strongly supports training and development of community-engaged scholars | 48% | 7% | 19% | 26% |  |
| Supports researchers and staff to learn from community partners | 44% | 6% | 16% | 34% |  |
| Encourages researchers to engage in health equity research | 19% | 16% | 44% | 22% |  |
| **Advocacy for Community or Patient-Engaged Research** |  |  |  |  |  |
|  | Strongly/  somewhat disagree | Neither agree nor disagree | Somewhat/Strongly agree | Don’t know |  |
| I am, or researchers I work with, are able to reflect and identify systems of power that influence treatment of community members and patients | 10% | 19% | 61% | 10% |  |
| I am, or researchers I work with, are able to negotiate for equitable services and research on behalf of community members/patients | 31% | 16% | 44% | 9% |  |
| I, or the researchers I work with, evaluate the effectiveness of efforts to address racial equity issues that impact community members and patients. | 16% | 19% | 63% | 3% |  |
| I am, or the researchers I work with, are comfortable developing an action plan to confront barriers to health equity that impacts community members and patients. | 6% | 22% | 59% | 13% |  |

*Note*. Similar response options were combined for presentation purposes.
